# Systematic evaluation of spatial resolution and gamma criteria for quality assurance with detector arrays in stereotactic radiosurgery

**DOI:** 10.1002/acm2.14274

**Published:** 2024-01-24

**Authors:** Ann‐Kathrin Stedem, Mark Tutty, Ndimofor Chofor, Marco Langhans, Christoph Kleefeld, Andreas A. Schönfeld

**Affiliations:** ^1^ Asklepios Klinik St. Georg Hermann‐Holthusen‐Institut für Strahlentherapie Hamburg Germany; ^2^ Radiotherapy Department Beacon Hospital Dublin Ireland; ^3^ Physics Research and Outreach Sun Nuclear A Mirion Medical Company Norderstedt Germany; ^4^ Klinik für Strahlentherapie Wilhelmshaven Germany; ^5^ National University of Ireland School of Physics University Road Galway Ireland

**Keywords:** detector arrays, gamma index, quality assurance, stereotactic radiosurgery

## Abstract

**Purpose:**

To characterize detector array spacing and gamma index for quality assurance (QA) of stereotactic radiosurgery (SRS) deliveries. Use the Nyquist theorem to determine the required detector spacing in SRS fields, and find optimal gamma indices to detect MLC errors using the SRS MapCHECK, ArcCHECK, and a portal imaging device (EPID).

**Methods:**

The required detector spacing was determined via Fourier analysis of small radiation fields and profiles of typical SRS treatment plans. The clinical impact of MLC errors of 0.5, 1, and 2 mm was evaluated. Global gamma (low‐dose threshold 10%) was evaluated for the three detector systems using various combinations of the distance to agreement and the dose difference.

**Results:**

While MLC errors only slightly affected mean dose to PTV and a 2 mm thick surrounding structure (PTV_2 mm), significant PTV underdose incurred with increase in maximum dose to PTV_2 mm. Gamma indices with highest sensitivity to the introduced errors at 95% tolerance level for plans on target volumes of 3.2 cm^3^ (plan 3 cc) and 35.02 cm^3^ (plan 35 cc) were 2%/1 mm for the SRS MapCHECK and 2%/3 mm for the ArcCHECK, with 3%/1 mm (plan 3cc) and 2%/1 mm (plan 35cc) for the EPID. Drops in passing rates for a 2 mm MLC error were (46.2%, 41.6%) for the SRS MapCHECK and (12.2%, 4.2%) for the ArcCHECK for plan 3cc and plan 35cc, respectively. For Portal Dose, values were 4.5% (plan 3cc) and 7% (plan 35cc). The Nyquist frequency of two SRS dose distributions lie between 0.26  and 0.1 mm^−1^, corresponding to detector spacings of 1.9 and 5 mm. Evaluation of SRS MapCHECK data with doubled detector density indicates that increased detector density may reduce the system's sensitivity to errors, necessitating a tighter gamma index.

**Conclusions:**

The present results give insight on the performance of detector arrays and gamma indices for the investigated detectors during SRS QA.

## 1 INTRODUCTION

In stereotactic radiosurgery (SRS) deliveries, slight misalignment of the treatment field or inaccuracies in the positioning of the multileaf collimators (MLCs) could result in clinically significant deviations in dose to the planning target volume (PTV) and organs at risk (OAR). The clinical impact of MLC errors have been widely studied for a variety of modern radiotherapy delivery techniques,^1^, ^2^, ^3^ highlighting the important role of patient specific quality assurance (PSQA).^4^ The success of the dose verification process is highly dependent on the ability of utilizing the dosimetric system to detect clinically relevant errors, while minimizing or avoiding any false positives.^5^ Two‐dimensional diode‐based detector‐arrays have proven to be very reliable, showing less variation in passing rates for error‐free plans as compared to widely used radiochromic film dosimeters.^6^ Additionally, detector arrays perform real‐time dose read‐out and are less complex to use than dosimetric films. Detector arrays, however, have a lower spatial resolution than film, due to the relative size of the single detectors and the inter‐detector spacing.^4^, ^7^, ^8^ To ensure that the SRS dose distributions are recorded by the detector array without any relevant information loss, the Nyquist‐Shannon sampling theorem should be fulfilled. This requires that the spatial resolution of the array must be to less than or one‐half the highest spatial frequency of the sampled SRS dose distribution.

In the gamma index analysis method,^9^ passing rates depend on influencing factors including the normalization method, the noise in the data sets, the choice of the reference dose distribution, and the resolution of the comparative distributions.^4^, ^10^, ^11^ During PSQA verifications, the gamma criteria should be systematically chosen since they depend on the detector used.^12^ Therefore, detector and even treatment unit dependent action limits (AL) and tolerance limits (TL) must be determined for error detection and troubleshooting.^13^ Recent recommendations have been made regarding selecting the gamma criteria used for dose verification of IMRT plans.^4^ They recommended for IMRT deliveries to use global normalization with a 10% dose threshold and a 3%/2 mm metric, with a 95% TL and 90% AL. For stereotactic ablative radiotherapy deliveries, the tighter 2 %/1 mm metric was shown in another study over a 2‐year period to be very versatile for detecting possible setup errors.^14^

Gamma indices for cranial stereotactic deliveries are investigated in this work using the onboard electronic portal imaging device (EPID) aS1200 with Portal Dosimetry (Portal Dose Image Prediction version 15.6.03, Varian Medical Systems, Palo Alto, California, USA), a planar radiation detector array commonly used in SRS QA, the SRS MapCHECK, and the cylindrical detector array ArcCHECK (both Sun Nuclear, a Mirion Medical Company, Melbourne, Florida, USA). In order to give more insight and guide the selection of QA devices and suitable gamma indices for the detection of clinically relevant errors, we structured this work into three phases, namely, (i) evaluate the clinical impact of small MLC errors from 0.5 to 2 mm, (ii) use signal theory analysis to determine the required detector resolution for accurate reconstruction of SRS radiation fields, and (iii) to investigate the sensitivity of three detector systems and optimal gamma analysis metric for the detection of MLC errors during SRS deliveries. This shall give more clarity to the counter‐intuitive observation that an increased resolution of a two‐dimensional detector array system does not always translate into an increase in error detectability. Results shall offer insight in selecting tools and guide recommendations on choosing optimal gamma analysis settings during SRS QA.

## 2 MATERIALS AND METHODS

To the best of our knowledge, no prior study has comprehensively integrated signal theory analysis with 2D detector array resolution for MLC error detection. We systematically assess the clinical impact of MLC errors on SRS plans and determine the required detector resolution using signal theory. Additionally, we create a standardized evaluation framework for various 2D detector types and treatment plans to detect these errors.

### 2.1 Clinical impact of MLC misalignments in SRS dose deliveries

The clinical impact of MLC errors was evaluated by systematically introducing leaf position errors and analyzing the effects on the dose volume histograms (DVH) for the PTV and the OARs. Using the Eclipse treatment planning system (TPS) version 15.6.04 (Varian Medical Systems, Palo Alto, USA) with 6 MV flattening filter free (FFF) photon beams, optimization was performed with the Photon Optimizer and the AAA calculation algorithm (both version 15.6.04). Two clinical cranial coplanar volumetric modulated arc therapy (VMAT) plans, each consisting of four arcs were investigated. The first plan (plan 3cc) was generated for two cranial PTV's with a combined PTV volume of 3.2 cm^3^ and planned with a single isocenter. Its delivery was intended for one fraction at 20 Gy with two full arcs and two half arcs and a total of 8511 monitor units (MU). Planning was completed with 100% of the PTV receiving at least 20 Gy with a maximum dose of 32.54 Gy within the PTV. The second plan (plan 35cc) only had a single but larger PTV of 35.02 cm^3^ volume, delivering 27 Gy in three fractions with 2973 MU per fraction. The planning was completed with at least 98% of the PTV receiving 98% of the prescribed dose. Both plans are representative of clinical SRS plans.

**FIGURE 1:**
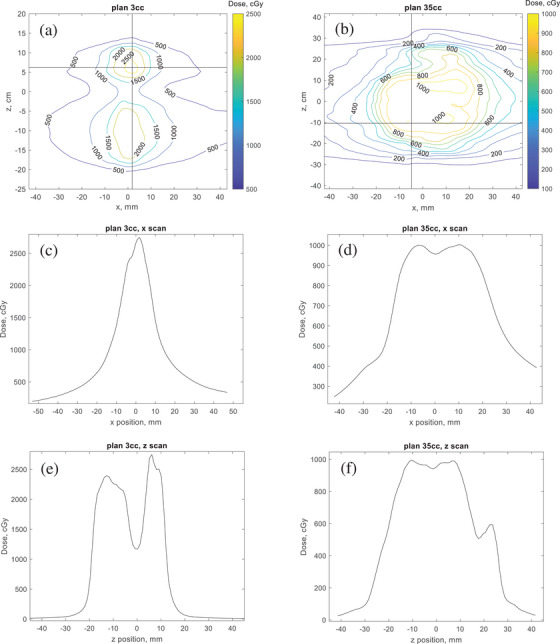
Dose maps of the cumulative dose of the VMAT QA plans, calculated on the StereoPHAN phantom, for plan 3cc (a) and plan 35cc (b) at the isocenter plane. Indicated scan profiles along the x‐axis (panels c and d) and z‐direction (e and f) were extracted for gamma analysis.

For dosimetric QA with the SRS MapCHECK, Figures 1a and b show corresponding dose maps at the isocenter plane, calculated in the TPS within the StereoPHAN. Dose profiles are shown for lateral scans (x‐axis) in panels (c) and (d) for longitudinal scans (z‐axis) in panels (e) and (f). These profiles shall be used later for signal theory analysis and gamma evaluations. The calculation grid size for both plans was set at 1 mm. The original unmodified plans were compared with plans containing MLC errors of varying magnitudes. Plans were exported to an in‐house Python‐based code to introduce artificial MLC errors^15^ by closing the entire leaf bank by shifts of −0.5 , ‐1, and ‐2 mm and then reimported for final calculation into the TPS. In the contouring module of the TPS, a 2 mm margin was generated around the PTV to create a new structure PTV_2 mm, to assess the dose to a nearby OAR. Resulting DVHs from these calculations in the TPS were analyzed.

### 2.2 Theoretical and experimental validation of detector resolution requirements for the QA of SRS deliveries

The purpose of any detector system intended for the QA of SRS deliveries is its ability to flawlessly reconstruct dose patterns and detect any clinically relevant errors. With increased use of high gradient dose deliveries to enhance tumor control at tight boundaries to OARs, two underlying questions for this analysis are (i) what are the resolution limits for the use of already‐existing detectors and (ii) are new commercial devices practical to ensure an improved quality for pre‐treatment QA. To answer these questions, we determined the required sampling rate for complete reconstruction of typical dose patterns in SRS fields. According to the Nyquist‐Shannon sampling theorem, the detector spacing must be less than or one‐half the highest frequency of the sampled dose distribution. Open rectangular fields with sizes ranging from 5 × 5 mm^2^ to 40 × 40 mm^2^ and line dose profiles from Figure 1 were investigated to determine the sampling frequency for an error‐free reconstruction of the profiles. The data sets were imported into Matlab (version R2020a) and the integrated Curve Fitting toolbox was used to create fit functions for each profile. First, a chosen step width was used to discretize and vectorize the data prior to application of the fit function, to derive the dose vector for spectral analysis. The dose vectors of the different field sizes were adjusted to the same length by zero padding and transformed into the spectral space using a Fast Fourier Transform (FFT). The Nyquist frequencies were determined by examining at which sampling frequency aliasing effects could be detected in the spectrum. These effects occurred below a low cut‐off amplitude of 0.005, which was set to exclude high frequency noise components of the spectrum. To give more insight to guide detector selection for SRS QA, the theoretical coverage for the detector resolutions of the SRS MapCHECK, the ArcCHECK (both Sun Nuclear, a Mirion Medical Company, Melbourne, Florida), and the on‐board amorphous silicon‐based electronic portal imaging device (EPID) was modelled. The detector array systems investigated in this work have been detailly described in other publications.^16^, ^17^, ^18^ The major characteristic features are listed in Table 1.

**TABLE 1:** Array detectors evaluated in this work, alongside most relevant specifications.

Detector Array	Detector element (dimensions)	Detector spacing	Detector surface
SRS MapCHECK	SunPoint2 diode (0.48 × 0.48 mm^2^)	2.47 mm diagonal, 3.5 mm lateral	77 × 77 mm^2^
ArcCHECK	SunPoint diode (0.8 × 0.8 mm^2^)	10 mm	210 × 210 mm^2^
Electronic Portal Imager	aS1200 (0.336 × 0.336 mm^2^)	0.336 mm	430 × 430 mm^2^

Alongside the signal theory analysis, a simple test was performed to proof‐check the required detector resolution for complete dose profile reconstruction. Dose profiles were obtained for both plans as shown in Figure 1. Starting using dose profiles from the TPS calculated with 1 mm resolution, high‐resolution profiles were generated via interpolation to a 0.1 mm resolution, for more precise calculation of the gamma index,^19^ and used as reference. Subsequent less dense grids were used to obtain dose distributions in the same plane from the reference plan. This was done by sequentially omitting sampling points on the dose grid. A simplicial complex (abbreviated simplex) interpolation method^20^ was applied to the sparse dose patterns for comparison with the reference profile via 1D gamma analysis for DTA/DD 1%/1 mm. Simplex implements a line segment, triangle, or tetrahedra for one, two, or 3D dose distributions.

To guide recommendations for the selection of adequate gamma settings sensitive enough to detect these clinical errors, different combinations of DTA from 1 to 3 mm and DD of 1−3 % were investigated for measurements at a VARIAN TrueBeam STx linac equipped with HD120 MLC, using all detectors listed in Table 1, giving a total of nine different evaluation indices for each test plan, and introduced MLC error. A 10% low‐dose threshold was used for all gamma analysis in this work. The plans were delivered, with separate acquisition of each single arc. To minimize setup positional errors, the modified plans were delivered for each detector immediately after delivering the original plan without changing the setup. This way, the major difference between the deliveries, besides any uncertainties from the treatment unit, would be the artificially induced MLC error.

## 3 RESULTS

### 3.1 Clinical impact of MLC errors in SRS dose deliveries

**FIGURE 2:**
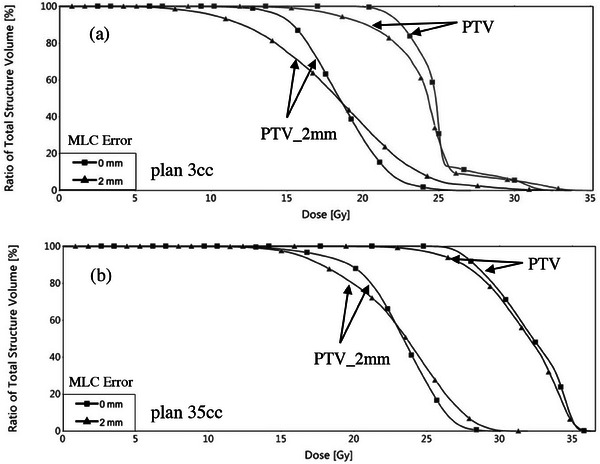
Dose volume histograms of plan 3cc (a) and plan 35cc (b) indicating the effect of the 2 mm MLC misalignments on the PTV and the neighboring structure PTV_2 mm.

Figure 2 shows the dose volume histograms from error‐free and modified plans computed in the TPS for, indicating the effects of the MLC errors on the PTV and the PTV_2 mm structures. As mentioned earlier, PTV_2 mm is simply used in this work to quantify the potential impact of dose changes on nearby OARs located adjacent to highly fluence modulated SRS fields. Given the smaller target volume in plan 3cc, the impact of the MLC misalignments on the PTV and PTV_2 mm are larger than in plan 35cc. Additionally, the severity of any clinical effects would comparatively be higher in plan 3cc, given that its delivery is intended for a single fraction only. The impact of the introduced MLC misalignments on the PTV and PTV_2 mm are shown in more detail in Table 2 for plan 3cc and in Table 3 for plan 35cc. The dose coverage to PTV and the mean and maximum doses to PTV and PTV_2 mm are shown alongside the percentage change from the original plan without misalignments in brackets.

**TABLE 2:** Effects of MLC misalignments on PTV and PTV_2 mm for plan 3cc (deviation from original plan).

MLC error	0 mm	0.5 mm	1 mm	2 mm
PTV: 98 % coverage	105.5 %	104 % (−1.5 %)	99 % (−6.5 %)	82 % (−23.5 %)
PTV: Min dose [Gy]	19.4	18.5 (−4.7 %)	16.8 (−13.3 %)	11.63 (−40.1 %)
PTV: Mean dose [Gy]	24.8	24.7 (−0.5 %)	24.7 (−0.6 %)	23.96 (−3.5 %)
PTV: Max dose [Gy]	32.5	33.8 (3.8 %)	34.3 (5.5 %)	35.18 (8.1 %)
PTV_2 mm: Mean dose [Gy]	18.5	18.6 (0.4 %)	18.5 (0.0 %)	18.15 (−1.9 %)
PTV_2 mm: Max dose [Gy]	27.1	31.3 (15.3 %)	32.7 (20.5 %)	33.75 (24.5 %)

**TABLE 3:** Effects of MLC misalignments on PTV and PTV_2 mm for plan 35cc (deviation from original plan).

MLC error	0 mm	0.5 mm	1 mm	2 mm
PTV: 98 % coverage	99.7 %	99.5% (−0.2 %)	97.7 % (−2.0 %)	90.1 % (−9.6 %)
PTV: Min dose [Gy]	24.3	24.2 (−0.6 %)	22.7 (−6.8 %)	18.5 (−24.0 %)
PTV: Mean dose [Gy]	32.0	31.9 (0.03 %)	31.9 (−0.3 %)	31.5 (−1.4 %)
PTV: Max dose [Gy]	36.3	36.0 (−0.6 %)	36.3 (−0.1 %)	36.6 (0.9 %)
PTV_2 mm: Mean dose [Gy]	9.07	9.1 (0.6 %)	8.4 (−6.9 %)	7.6 (−16.1 %)
PTV_2 mm: Max dose [Gy]	30.1	30.3 (0.8 %)	30.8 (2.5 %)	31.9 (6.2 %)

For plan 3cc, PTV coverage dropped significantly by up to 23.5% and by 9.6% for plan 35cc after systematically closing the MLCs by 2 mm. Consequently, the degree of PTV underdosage was worsened by a drop in the minimum dose to PTV by 40.1% and 24% for plan 3cc and plan 35cc, respectively. Following the same trend, the mean dose to the PTV each dropped successively with increased MLC error by up to 3.5% for plan 3cc and 1.4% for plan 35cc. The change in dose maximum to the PTV was larger for plan 3cc, with already 3.8% for the 0.5 mm MLC error and reaching 8.1% at 2 mm MLC shift. For plan 35cc, no significant change in the maximum dose to the PTV was calculated in this study. For the 2 mm MLC error, the maximum dose to the nearby structure PTV_2 mm increased by 24.5% and 6% respectively for plan 3cc and plan 35cc. This is shown more clearly in Figure 2. The clinical impact of the MLC errors become more significant as the PTV volume reduces.

### 3.2 Signal theory analysis of SRS deliveries and experimental validation

**FIGURE 3:**
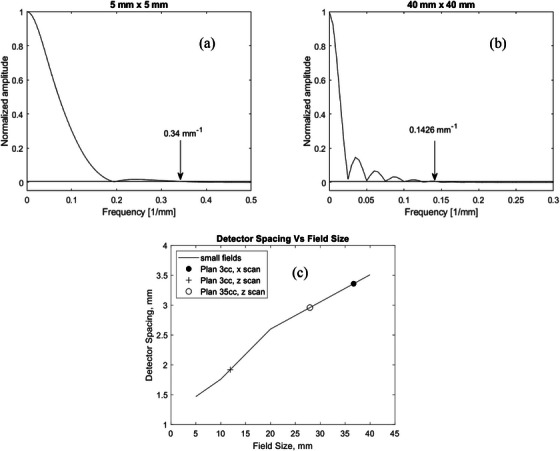
Fourier spectra of small field dose profiles, showing normalized amplitude spectra used for deriving the Nyquist sampling frequencies of radiation fields with field sizes of (a) 5 × 5 mm^2^ and (b) 40 × 40 mm^2^. (c) Dependence of the derived detector spacing on the field size. The detector spacing of 4.98 mm corresponding to the x‐profile of plan 35cc is not shown here.

**FIGURE 4:**
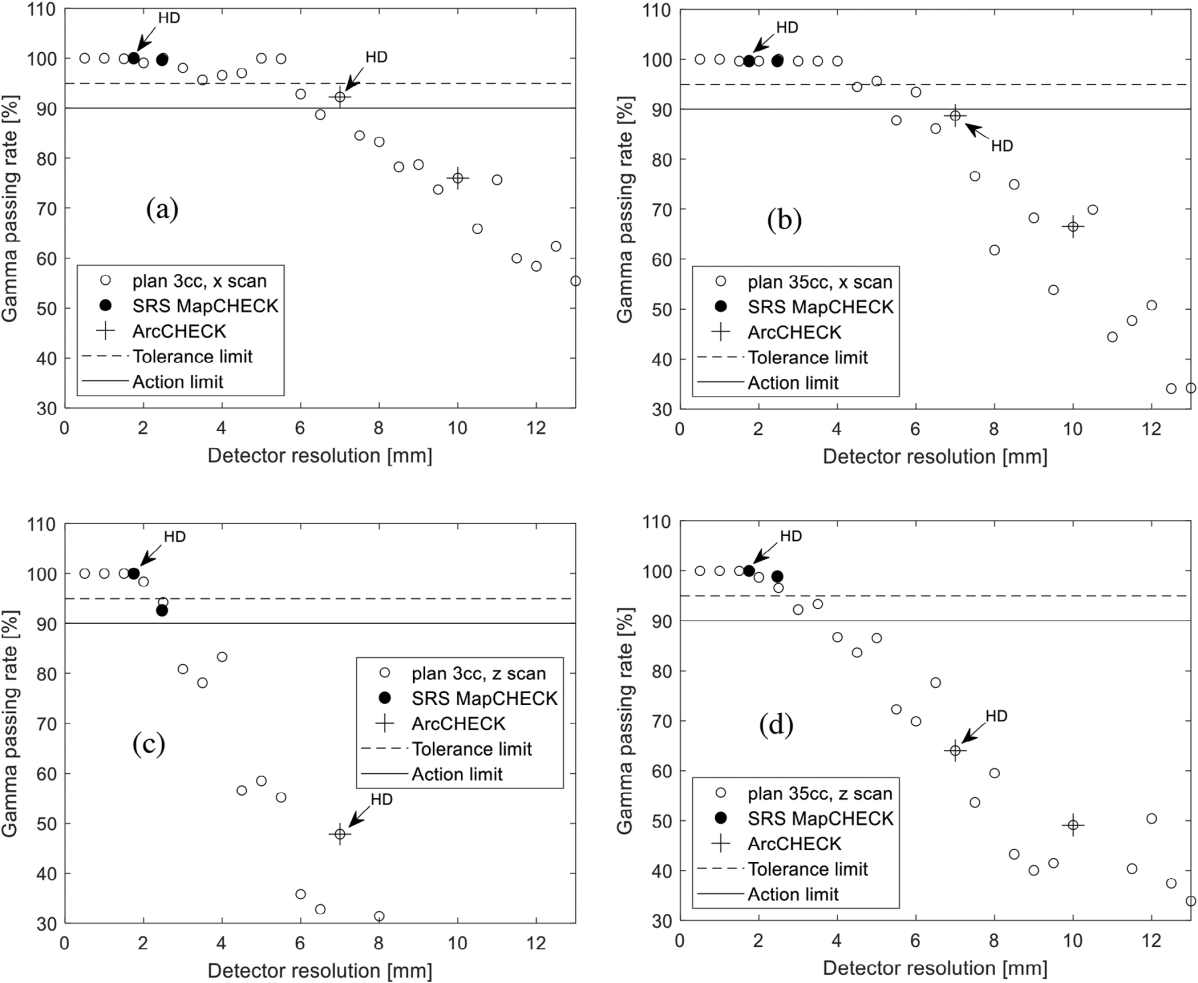
Comparison of the 1D gamma passing rates for DTA/DD 1 % / 1 mm for stepwise down‐sampled TPS dose profiles from plans 3cc and 35cc against the original datasets with 0.1 mm resolution (open circles). Resolutions corresponding to investigated detector arrays are highlighted for reference, including enhanced resolution measurement configurations (labeled HD).

Figure 3 shows exemplary results from the Fourier analysis of the small field dose profiles, alongside data points for the scanned profiles from Figure 1 for plans 1 and 2. In the frequency domain, the spatial frequency content of a dose profile is expressed in units of 1/mm and a specified amplitude. By setting the cut‐off at 0.005, the derived Nyquist frequencies ranged from 0.68 mm^−1^ for the 5 × 5 mm^2^ field, to 0.285 mm^−1^ for the 40 × 40 mm^2^ field. The corresponding detector spacings are 1.47 mm for 5 × 5 mm^2^ and 3.5 mm for the 40 × 40 mm^2^ field. Based on Fourier analysis of the x‐scanned dose profiles from plans 1 and 2, detector spacings were, respectively, 3.36 and 4.98 mm. For the z‐scans, the derived maximum detector spacings were 1.92 mm for plan 3cc and 2.96 mm for plan 35cc. The detector spacing for the data point for plan 35cc, x scan at 4.98 mm is not displayed. Data points are shown in the figure for didactic purposes only and should not be mistaken for the effective field size, this is not the subject of the present study. This gives a good insight into the field sizes corresponding to the determined detector resolution. The required detector spacing for a designated detector array would also depend on the lateral dose response function of the single detector elements.^8^ Given these effects and the inherent detector characteristics, a detector's performance in the verification of SRS deliveries can be tested using the gamma analysis. Figure 4 shows a general evaluation of the sampling rate required to represent the SRS dose distribution. The detector spacing for the two commercial detectors investigated in this study are also plotted, although actual passing rates would depend on physical factors during beam delivery such as the linac stability and detector type. For instance, air‐filled detectors have a wider response function compared to diodes.^21^ Nevertheless, resampling dense profiles sparsely to fit the grid spacings of the high‐density dose map would eventually lead to lower passing gamma rates. Also tested was a high‐resolution dataset, commercially integrated and obtainable for the SRS MapCHECK and ArcCHECK. This is performed using two subsequent measurements for the same treatment plan and applying a longitudinal detector shift of half the inter‐detector spacing between both measurements. For each detector, the high‐resolution version is the data point to the left in Figure 4.

### 3.3 Evaluation of different gamma indices

**FIGURE 5:**
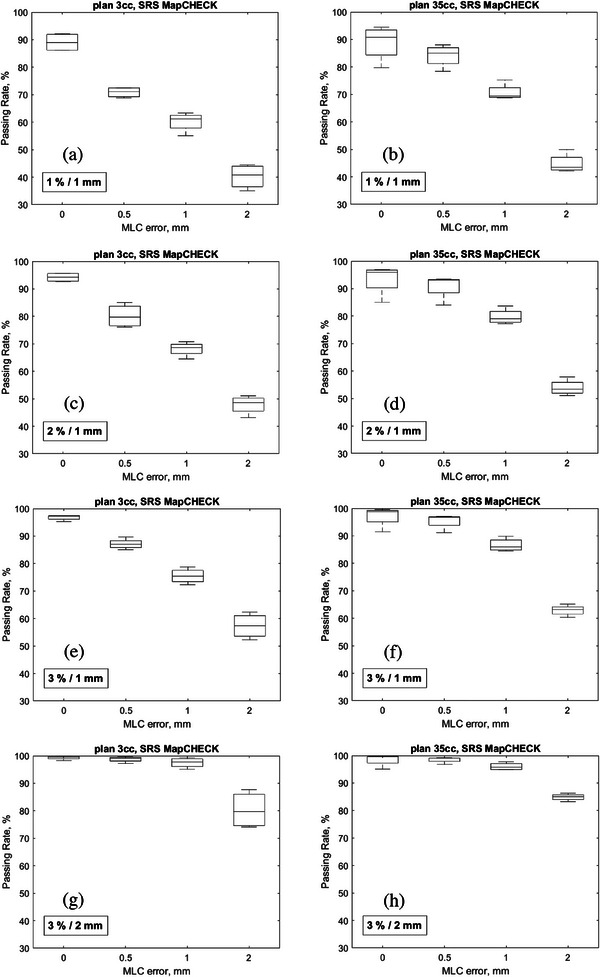
Gamma passing rates for various DTA/DD combinations for the SRS MapCHECK for plans 1 and 2. The 1%/1 mm metric is too strict and falls below the 90% action limit for both plans.

It is crucial to consider the specific characteristics of the detector and treatment unit in use when selecting the appropriate gamma passing rate and DTA/DD criteria. The dependence of the selected criteria on the linac and the detector system was elaborated in a retrospective study with 200 VMAT plan verifications using a MapCHECK 2 and ArcCHECK (both Sun Nuclear Corp., Melbourne, Florida, USA) on two different linacs.^13^ We thereby specify that the results presented in this work is applicable to the linac and detectors evaluated and must be validated for other linac/detector combinations. In Figure 5, box plots are shown for both plans with and without MLC errors, with the gamma pass rates separated into the various DTA/DD metric combinations evaluated for the SRS MapCHECK. The spread of the data, comprising of data from each measured arc, indicates the varying sensitivity of the different indices to detect the introduced MLC errors. The fluctuation in the data at 0 mm MLC error may be an indication of uncertainties from the treatment planning and beam delivery chain. Treatment planning system (TPS) beam model‐related uncertainties may result from inaccuracies during MLC modelling, measurement of leaf transmission, output factors, head scatter, and off‐axis profiles.

Given the potential clinical impact of small MLC errors during SRS deliveries, as demonstrated in the example in Section 3.1, it may be appropriate to set a 95% tolerance limit (TL) and a 90% action limit (AL) for the gamma index. During verification of the error‐free plans, a passing rate below 90% may be an indication that the chosen metric is too strict. The recommended metric for routine SRS QA should not be too strict and thereby show a pass for an unmodified plan. The 1 mm DTA metric combinations were the most sensitive to the MLC errors. While the 1%/1 mm metric was too strict and failed the error‐free plans, the 2% and 3% combinations with 1 mm DTA remained within range of the 95% passing rate and above the 90% limit. As such, the 1%/1 mm metric would be unreliable for clinical QA of SRS deliveries using the SRS MapCHECK. If we use the 95% TL, the metric combinations with 2  and 3 mm DTA would not be sensitive enough to detect the MLC errors using this device. The combinations with 2 mm DTA only fell below the 90% (AL) for the 2 mm MLC error.

**FIGURE 6:**
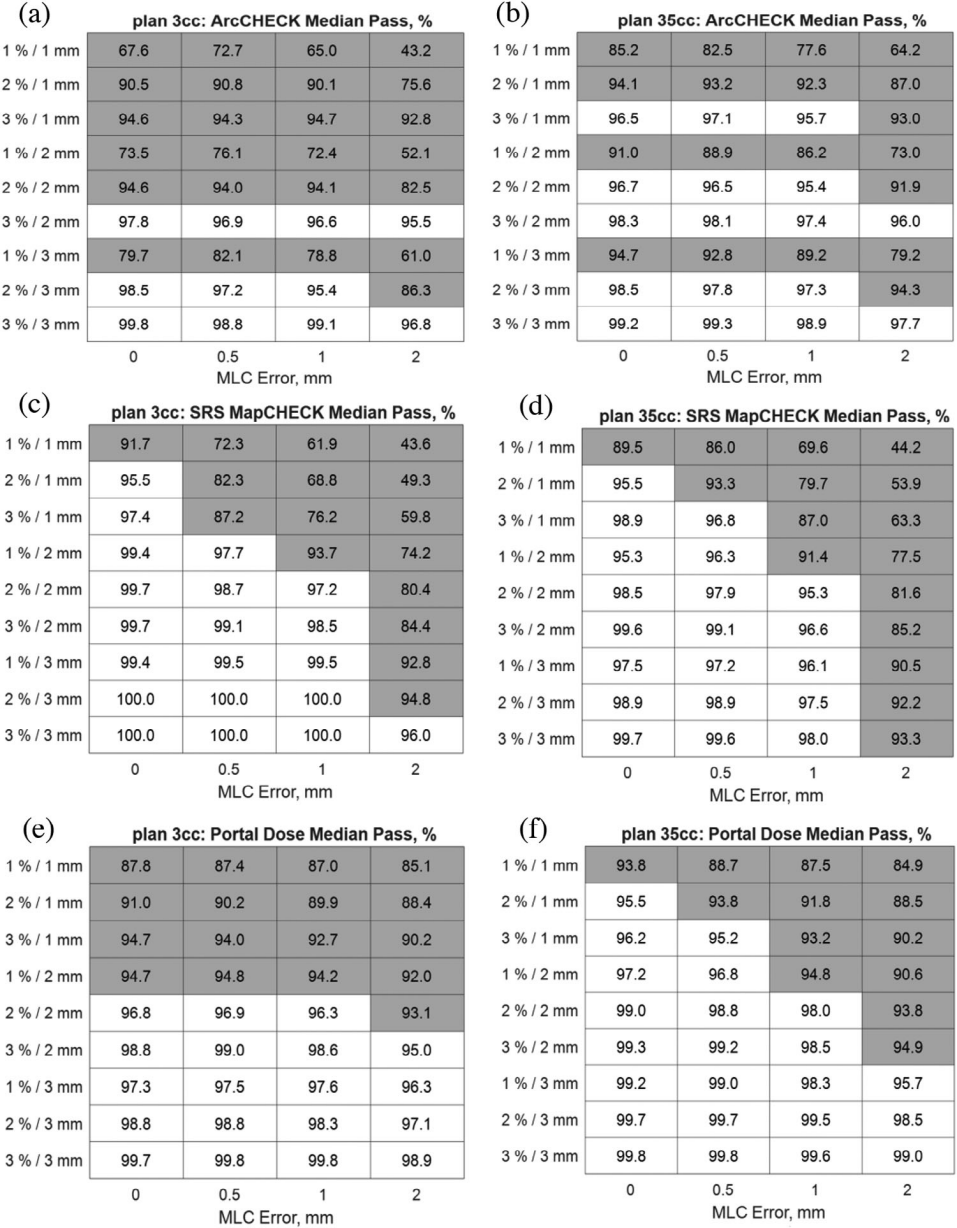
Median passing rates for the investigated gamma metrics and simulated MLC errors for plan verifications with ArcCHECK, SRS MapCHECK, and the onboard electronic portal imaging device (Portal Dose).

Although a similar trend was observed using the other investigated detectors, a separate analysis must be done for each detector system. Figure 6 summarizes results for the three detector array systems as shaded maps. The results below the passing rate of 95% are shown in the grey area. There is a fundamental interplay between the detector resolution and the sensitivity to introduced errors. For high resolution detector arrays, the dose difference DD plays a more significant role than the distance to agreement DTA. Following this rationale, data was ordered such that DD was varied for the same DTA. For the ArcCHECK by keeping DD constant and varying DTA the heat maps would best depict the trend for this lower‐resolution detector. For the sake of consistency in the display of the results, the values are sorted from lowest to highest, with fixed DTA at varying DD.

The similarity between the passing rates of the plans with 0, 0.5, and 1 mm MLC errors indicates the relative insensitivity of the ArcCHECK to detect these errors at 95% gamma passing rate. The general indication, supported partially by the results in Figure 4, is that the ArcCHECK in its standard detector resolution is not the preferred candidate for SRS QA. Preliminary results with the high‐density data merge showed no significant change of the pass rates. This is evident from the profiles in Figure 4, showing the passing rate in the standard and HD configurations for the ArcCHECK which still lie below the thresholds for the dose profiles evaluated in this analytic example. For the SRS MapCHECK, the 1%/1 mm metric is too strict, almost reaching the 90% threshold for plan 3cc and falls below this level for plan 35cc. For this detector, only the indices with DTA 1 mm were sensitive enough to detect the 0.5 mm MLC error. None of the DTA 3 mm combinations had a pass rate below 90% for the MLC errors in plans. For the EPID used with Portal Dosimetry, the results were generally inconsistent with the 1%/1 mm metric being too sensitive and failing without any MLC error in plan 3cc but detecting the errors in plan 35cc. Despite the introduced MLC errors, none of the other metric combinations beyond DTA 1 mm showed a passing rate lower than 90% for either plan. Though this may indicate that stricter indices should be used for detectors with high resolutions, it also shows the need for better characterization of the specificity of a designated detector system prior to its use in the verification of SRS deliveries. Since the rate of change of the Portal Dose results with respect to the MLC error magnitude is small, the interpretation of the measurements may be sensitive to the implemented algorithm, EPID performance, or set passing threshold. However, the results found in this work align well with other studies using different algorithms and a aSi 500 detector.^22^ While their median passing rates for intracranial IMRT deliveries with flattened beams were 99% for 3%/2 mm and 94.9% for 2%/1 mm, the values from this work with SRS deliveries were 99.3% for 3%/2 mm and 95.5% for 2%/1 mm. From Figure 6, gamma indices that maximized the rates of change of the passing rate with respect to the magnitude of the introduced error using a 95% tolerance level were 2%/1 mm for the SRS MapCHECK, 2%/3 mm for the ArcCHECK, and either 3%/1 mm (plan 3cc) or 2%/1 mm (plan 35cc) for the EPID. Corresponding drops in passing rates for a 2 mm MLC error were (46.2%, 41.6%) for the SRS MapCHECK and (12.2%, 4.2%) for the ArcCHECK for plan 3cc and plan 35cc, respectively. For Portal Dose, drop in passing rates were 4.5% for plan 3cc and 7% for plan 35cc.

## 4 DISCUSSION

MLC misalignment errors may have clinically significant effects, especially around the typical steep dose gradients surrounding often highly modulated radiotherapy fields. In a recent study dedicated to single‐isocenter multi‐metastatic SRS deliveries for lesions of sizes 0.46–4.42 cc, dose increases between 20% and 30 % were reported for nearby risk organs for a 1 mm MLC error,^1^ matching the 24% maximum dose increase reported in this work. While earlier research explored the relationship between detector resolution and the gamma passing rate,^23^ to the best of our knowledge, no study has integrated a signal theory analysis with the resolution of 2D detector arrays in a comprehensive context related to MLC error detection. We complement previous efforts to provide insights on selecting QA devices for verifying stereotactic plans, taking into account the increasing use of detector systems with resolutions as small as 0.4 mm. The required detector resolution (detector element spacing *d*) was evaluated using Fourier analysis of small fields and scanned dose profiles from SRS plans. Complementary evaluation was also performed using resampled treatment plan profiles to demonstrate the use of detector element spacing on the gamma pass rate. In order to perform inter‐comparison of reference and measured dose profiles, the grid spacings must match. In a paper on the implementation of a fast 3D gamma evaluation method, a grid spacing of less than 1/10 of the DTA parameter was recommended for a precise calculation of the gamma index.^19^ The matching of data points usually requires interpolation, whereby the accuracy of the interpolation may in turn influence the accuracy of the determined gamma index. In this work, gamma analysis was performed by representing the dose surface of the evaluated distribution using a simplicial complex.^20^ This method is also commercially available with the SRS MapCHECK and ArcCHECK devices. The results from Figure 4 indicate the theoretical limits of the required detector resolution based on the distribution of the dose alone. The required detector resolution is a combination of the required dose sampling rate and the characteristics of the single detector elements. In previous work on an air‐filled ionization chamber array,^8^ the requirements for the detector spacing were shown to decrease, compared to an array with diodes. This comes from the signal smoothing effect correlated with the wide lateral response of the air‐filled chamber, which extends beyond the comparably larger detector boundaries. The lateral response function of point‐shaped diodes is zero outside the boundaries, so that a diode array system picks up an unsmoothed signal distribution, which may require a smaller inter‐detector spacing compared to an ionization chamber array.

**FIGURE 7:**
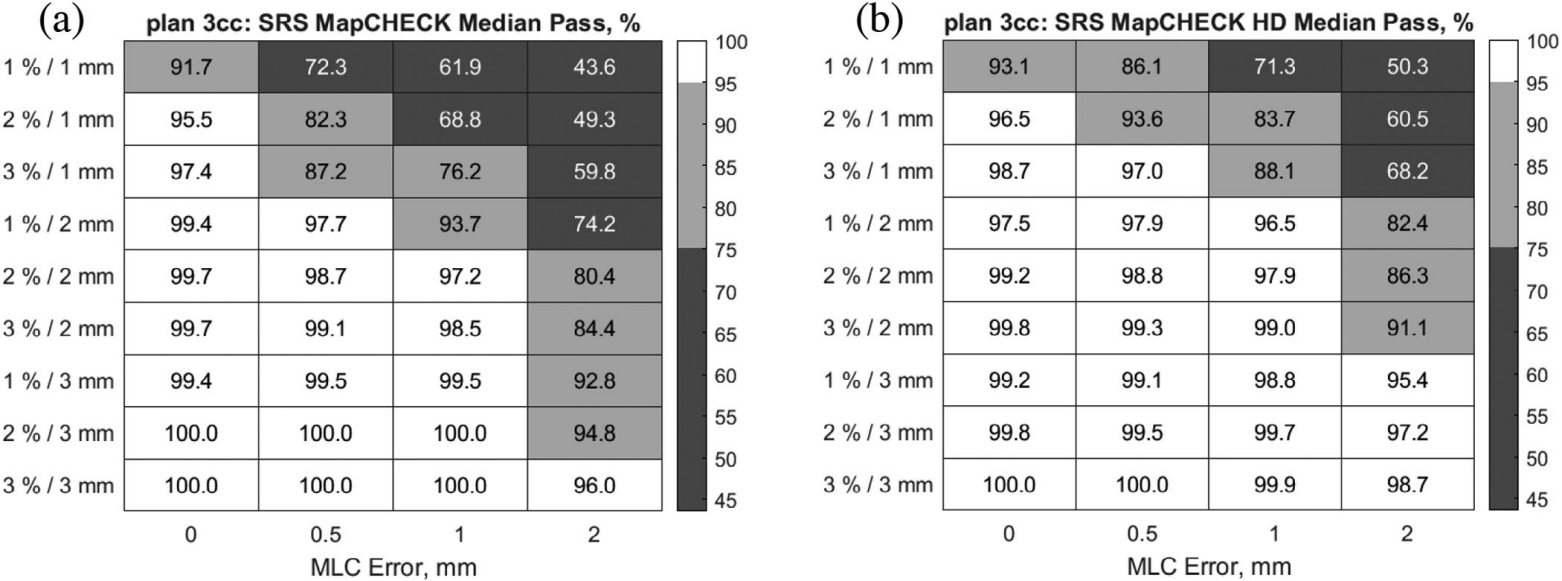
Median passing rates for the investigated gamma metrics and simulated MLC errors measured with the SRS MapCHECK for plan 3cc, using (a) the standard detector resolution and (b) the high‐density (HD) resolution mode.

**FIGURE 8:**
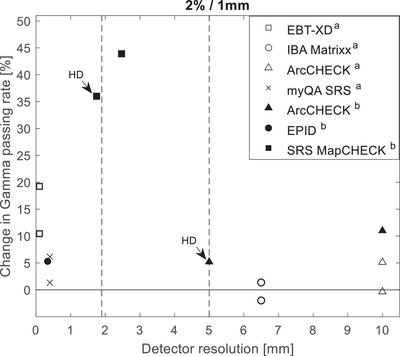
Change in 2%/1 mm gamma pass rates versus array detector spacing for 2 mm MLC errors. Vertical dotted lines show the required detector spacing for correctly sampling the SRS dose profiles derived in this study using the Nyquist theory. Open symbols show data from other authors, for lesions of volumes 35.81–161.13 cc.^23^ For each detector resolution, superior datapoint for stuck leaf and inferior datapoint for lagging leaf error. Filled symbols show data from this study.

A gamma analysis of SRS plans with and without MLC errors was used to guide the use of the DTA/DD combinations in this work and complement the theoretical analysis of the required detector resolutions. Depending on the treated site and modality, the choice of DTA/DD would play a significant role in the decision‐making regarding passing criteria for radiotherapy plan deliveries and must thereby be chosen cautiously. By comparing the gamma passing rates for DTA/DD 1%/1 mm an extreme scenario was evaluated using the steep‐dose‐profile z‐scan from plan 3cc (see Figure 1). This result may be misleading, and one may think that the detector resolution of the SRS MapCHECK may be insufficient when measuring with the standard detector density and raises the question if there is an effect on the error detection capability. To further clarify this, we delivered plan 3cc twice and performed a high‐density (HD) merge by shifting the detector longitudinally by one‐half the detector spacing, i.e., by 1.75 mm between both measurements for complete field coverage. Results are shown in Figure 7 for the standard resolution (a) and high‐resolution (b). From this intriguing observation, the dependence of the change in passing rate of gamma 2%/1 mm on the detector spacing is shown in Figure 8. Data from another study on SRS deliveries on target volumes of sizes 0.43–161.13cmm is also included to depict the trend.^24^ This trend is indicative of the complexity behind the influencing factors, which may include treatment plan and delivery as well as measuring technique. Figure 8, however, helps underline the role of the Nyquist theory on MLC error detectability, whereby an optimal detector spacing should preferably lie within the boundaries defined by the vertical dotted lines.

One would expect that this high resolution would improve the error detection capacity of the system. In fact, the SRS MapCHECK HD analysis of the full plan shows that increasing detector density would require the stricter 1%/1 mm metric to detect MLC errors. The increased resolution may thereby mask potentially clinically relevant effects when less strict DTA/DD indices are used. A likely reason for this effect is the fact that regions in the dose plane that require the highest resolution are very small and MLC errors will be more conspicuous in the large regions of the dose planes. However, since with a higher resolution array more detectors are in unaffected regions of the computed dose volume, the passing rate is artificially enhanced, an effect previously reported in another study investigating the dependence of gamma pass rates for SBRT plans using three 2D array detectors used in normal and increased detector density.^23^ This effect is analogous to 2D versus 3D gamma analysis. Since the selection of the gamma indices should also consider external factors, such as setup uncertainties of the phantom and signal noise, and the gamma index thereby have a practical lower boundary, it can be concluded from this evaluation that the array resolution in the HD setup can indeed be too high. An attempt was made to demonstrate this effect using the high‐resolution CMOS‐based detector myQA SRS (IBA Dosimetry, Schwarzenbruck, Germany) and systematically perform gamma analysis on down‐sampled data. Unfortunately, a burn‐in ghost image was observable in the background reading during deliveries of the four versions of both test plans. On a second attempt, the measurement results of rotational deliveries were off by 30%, which was assumed to be due to incorrectly applied angular corrections. This led to inconsistencies in the dose output given for instance the high cumulative dose of 80 Gy delivered for variations of plan 3cc alone. Coined with the limited availability of the device for further tests, results were omitted in this work and a separate dedicated study is planned to address these issues. However, the results for the myQA SRS and radiochromic film cited in Figure 8 support the observation made in Figure 7, i.e., an excessive detector array resolution can result in a decreased error detection capability. Regarding the EPID results, the measurement method is fundamentally different from stationary arrays, i.e., the EPID accumulates the fluence map in a perpendicular composite style. Hence, small changes in the fluence map induced by the MLC errors only affect a small portion of the total fluence map recorded by the EPID. Consequentially, the passing rate is always quite high and only changes marginally with increasing MLC error magnitude, as best visible in Figure 6e. The error sensitivity appears to be further reduced for the smaller lesion with a difference of only about 4.5% for plan 3cc and of about 7% for plan 35cc. This drop in the Portal Dose error sensitivity with decreasing lesion size has also been reported in other studies.^25^

Given the correlation between the gamma index and detector element spacing *d*, for the ArcCHECK (*d* = 10 mm) for instance, Figure 6 indicates the limits of utilizing this device for SRS QA. The 2%/2 mm and 2%/3 mm indices could only detect 2 mm MLC errors. These indices are, however, relatively insensitive to slight MLC errors when using the high‐resolution SRS MapCHECK (*d* = 2.47 mm). For the SRS MapCHECK, the 2%/1 mm index was sufficiently sensitive for the detection of clinically relevant MLC misalignments, and not too strict to fail error‐free plans. This result is consistent with a similar recent study comparing various detector arrays against film dosimetry.^24^ The Fourier analysis in the present work (see Figure 3) shows that the detector spacing in the SRS MapCHECK is sufficient for the investigated SRS fields. This highlights the importance of a small distance to agreement (DTA) value for high‐resolution detector arrays when altering the dose difference (DD) to detect errors.

## 5 CONCLUSION

In this work, we evaluated the potential clinical impact of small MLC misalignments as low as 0.5–2 mm during stereotactic radiosurgery deliveries on the PTV and nearby OARs. While the mean doses to the PTV and OARs stayed almost constant with these introduced in‐field MLC errors, the underdosage of the PTV and dose maximum to the OARs changed significantly. From the Fourier analysis of small fields and SRS dose profiles, the required detector spacing for the correct measurement of the profiles lie between 1.9  and 5 mm. With this hindsight, a systematic evaluation of the MLC error detectability was tested for two commercially available array detectors alongside the onboard electronic portal imaging device (EPID) for various DTA/DD combinations.

Though the present study evaluated multiple systematic MLC positioning errors on two SRS plans: plan 3cc (2 PTVs with combined volume of 3.2 cm^3^) and plan 35ccm (single 35 cm^3^ PTV), further justification of the detection thresholds for the various detectors can be made by extending the investigations to other linacs and delivery techniques. We generally expect similar results from different delivery and planning systems provided the produced dose distribution feature similar spatial frequencies (dose gradients). To validate that assumption, the study extends to the extreme case of small, static, open fields that have steeper lateral dose gradients than produced by rotational delivery techniques, see Figure 3. The results presented here provide guidance on selecting a reasonable gamma DTA/DD analysis index for the investigated detectors during SRS QA. Portal Dosimetry, utilizing the onboard EPID detector, could detect the introduced MLC errors using the 3%/1 mm index in plan 3cc and the stricter 2%/1 mm index for plan 35cc. The ArcCHECK system, when used detected the 2 mm MLC errors most reliably with the 2%/3 mm index. Considering the trend towards increased accuracy in SRS deliveries nowadays, coined with the advent of detector array systems with higher resolutions, we recommend using stricter indices for QA. From the systematic evaluation of combinations of DD 1% to 3% and DTAs of 1 to 3 mm, the results indicate that the 2%/1 mm index would be sufficiently sensitive to detect clinically relevant MLC misalignments as low as 0.5 mm during SRS deliveries when using the SRS MapCHECK. Evaluation of a high‐density merge of SRS MapCHECK data indicate that an excessive detector density does not improve the error detection capability of an array, but instead requires a stricter gamma index to account for an increased number of passing detectors in non‐critical dose regions.

## AUTHOR CONTRIBUTIONS

All authors in this manuscript in one more of the following ways: study design and development, data collection and analysis, results and conclusion synthesis, and editing/revising. The first three authors A‐K. Stedem, M. Tutty, and N. Chofor equally share first authorship. A‐K. Stedem and M. Tutty performed all measurements and calculations. N. Chofor did data analysis and manuscript writing. M. Langhans and C. Kleefeld coordinated clinical work, alongside A. Schönfeld who also co‐reviewed manuscript editing/revising.

## CONFLICT OF INTEREST STATEMENT

The author declares that there is no conflict of interest that could be perceived as prejudicing the impartiality of the research reported.
